# Investment in sensory structures, testis size, and wing coloration in males of a diurnal moth species: trade-offs or correlated growth?

**DOI:** 10.1002/ece3.1459

**Published:** 2015-03-17

**Authors:** Brett P Shiel, Craig D H Sherman, Mark A Elgar, Tamara L Johnson, Matthew R E Symonds

**Affiliations:** 1 Centre for Integrative Ecology, School of Life and Environmental Sciences, Deakin University221 Burwood Highway, Burwood, Melbourne, Victoria, 3125, Australia; 2 Centre for Integrative Ecology, School of Life and Environmental Sciences, Deakin UniversityPigdons Road, Waurn Ponds, Victoria, 3217, Australia; 3 School of BioSciences, University of MelbourneMelbourne, Victoria, 3010, Australia

**Keywords:** Allometry, antenna size, coloration, Lepidoptera, receiver, sexual selection, signaling, testis size

## Abstract

For dioecious animals, reproductive success typically involves an exchange between the sexes of signals that provide information about mate location and quality. Typically, the elaborate, secondary sexual ornaments of males signal their quality, while females may signal their location and receptivity. In theory, the receptor structures that receive the latter signals may also become elaborate or enlarged in a way that ultimately functions to enhance mating success through improved mate location. The large, elaborate antennae of many male moths are one such sensory structure, and eye size may also be important in diurnal moths. Investment in these traits may be costly, resulting in trade-offs among different traits associated with mate location. For polyandrous species, such trade-offs may also include traits associated with paternity success, such as larger testes. Conversely, we would not expect this to be the case for monandrous species, where sperm competition is unlikely. We investigated these ideas by evaluating the relationship between investment in sensory structures (antennae, eye), testis, and a putative warning signal (orange hindwing patch) in field-caught males of the monandrous diurnal painted apple moth *Teia anartoides* (Lepidoptera: Lymantriidae) in southeastern Australia. As predicted for a monandrous species, we found no evidence that male moths with larger sensory structures had reduced investment in testis size. However, contrary to expectation, investment in sensory structures was correlated: males with relatively larger antennae also had relatively larger eyes. Intriguingly, also, the size of male orange hindwing patches was positively correlated with testis size.

## Introduction

Sexual selection favors the evolution of secondary sexual traits associated with mating success (Andersson 1994). Studies of underlying processes typically focus on traits such as weaponry or elaborate signals, which are associated with intrasexual competition and intersexual mate choice, respectively. In contrast, Darwin's (1871, p. 418) prediction that sensory structures should also become elaborate as a result of sexual selection has been largely ignored (for a rare example see Gwynne and Bailey 1999). The antennae of some species of moth are strikingly sexually dimorphic, with males possessing large feathery bipectinate antennae in comparison with the simpler antennae of females. Male antennae carry a much higher number of sensilla (Chapman 1982), which detect the minute quantities of sex pheromones released by the female (Svensson 1996). Larger, more elaborate male antennae are associated with species of moths that exist at low population densities, suggesting that sexual selection favors traits facilitating the rapid detection of mates that are highly dispersed and difficult to locate (Symonds et al. 2012). In diurnal moths, mate location by males may also involve visual searching (Charlton and Cardé 1990; Sarto i Monteys et al. 2012), and hence selection may also favor augmented eye size in males.

Elaborate traits are costly (Andersson 1994), and if two or more structures require investment from a shared resource pool, allocation trade-offs may arise, with the enlargement of one structure possible only at the expense of another (Reznick 1985; Bonduriansky and Day 2003). This is particularly relevant to holometabolous insects where the development of adult characteristics takes place after resource acquisition has ceased (Tomkins et al. 2005). Sensory structures in particular represent costly investments for insects, and investment in such structures may be reduced if resources are limited (Niven and Laughlin 2008). Several studies of insects have uncovered links between rearing conditions (e.g., diet quality, population density) and investment in antennae independent of body size, suggesting clear costs to these structures (e.g., *Gerris* water striders – Arnqvist and Thornhill 1998; *Teleostylinus* flies - Bonduriansky 2007a; *Polistes* wasps – Sheehan and Tibbetts 2011; but see Bonduriansky and Rowe 2005 for an example of no apparent link in *Prochyliza* flies). Consequently, given finite and possibly limited resources, investment in sensory structures may be traded-off against each other, or against other structures. For example in the horned dung beetle, *Onthophagus taurus*, larger horn size, important in competition between males, is associated with reduced eye size and antennal size (Nijhout and Emlen 1998; Emlen 2001).

For polyandrous species, trade-offs may also occur between pre-insemination (mate searching, male competition) and postinsemination (paternity protection) traits. In such species, investment in male structures associated with mating may occur at the expense of investment in testes. For example, in both the horned dung beetle *Onthophagus binodis*, and the weta *Hemideina crassidens*, males with larger horns or mandibles (used as weaponry in male-male competition) have smaller testes and ejaculates (Simmons et al. 1999; Kelly 2008). Because of the likelihood of sperm competition, polyandrous species typically invest more in sperm production, having larger testes and better sperm swimming mobility compared with monandrous species (Gage 1994). Trade-offs between pre- and postinsemination sexually selected traits are not predicted in monandrous species, as paternity protection is unlikely to be important and investment in testes can be minimized. Nevertheless, males may still trade-off investment in traits that facilitate mate location with those that favor survival. For example in the geometrid moth, *Epirrita autumnata*, males with larger wings (and hence improved dispersal ability) are more likely to locate females, but have reduced survival, at least at low temperatures (Tammaru et al. 1996).

**Figure 1:**
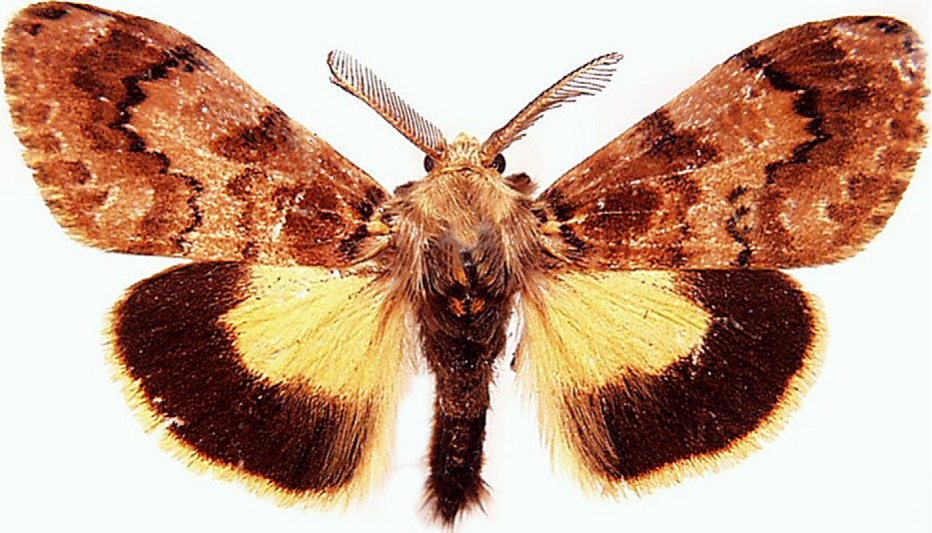
The male painted apple moth (*Teia anartoides*). Photograph reproduced by kind permission of Len Willan, CSIRO Entomology, www.csiro.au/resources/Australian-Moths.html.

Here, we test predictions in regard to trade-offs in males of a monandrous moth species, the painted apple moth *Teia anartoides* (Fig.1). This species is highly sexually dimorphic, with males possessing elaborate feathery antennae, while the apterous females have thin pinnate antennae. Unusually for moths, males of this species are diurnally active, and while typically cryptic in coloration at rest, have distinctive bright orange patches on their hindwings that are visible only during flight and mating. The role of these color patches is unknown, but it is unlikely that the orange coloration is a signal used in female choice because adult females are unable to resist male attempts at copulation (T. Johnson, B. Shiel, per. obs.). The most likely possibility is that the patches act as a warning signal to predators (other cryptic lepidopteran species have similar warning patches found only on their “hidden” hindwings: e.g. Forsman and Merilaita 2003). Thus, investment in these patches may reduce the likelihood of attack by predators during mate location. However, the expression of these warning colors is likely to be costly. In animals generally, the size and intensity of warning coloration imposes potential costs, both in terms of investment and in terms of conspicuousness (Stevens and Ruxton 2012). While most research on the condition-dependence of coloration in Lepidoptera has focussed on sexually selected coloration (Morehouse and Rutowski 2010; Tigreros 2013), there is also evidence that diet quality can determine the size and intensity of warning color patches (Lindstedt et al. 2010; Pegram et al. 2013), which suggests a cost that may come at the expense of investment in other traits such as sensory structures.

Here, we examine the natural variation in antennae size, eye size, testis size, and orange hindwing patch in *Teia anartoides*. We predict a trade-off (i.e., negative correlation) investment in male sensory structures (antennal size vs. eye size), but do not predict that these traits will be traded-off against testis size. We predict that total investment in male sensory structures may nevertheless be traded-off against investment in warning colors.

## Methods

### Field collection

We collected males from field sites in the eastern suburbs of Melbourne, Australia, using delta traps (Conservation Resources Pty Ltd, Enmore, NSW, Australia) baited with live females that had been reared from a laboratory culture. Traps were hung at 1–2 m height from trees during daylight hours (starting at between 7 and 9 am) and checked at the end of each day (4–6 pm). In total, 51 captured males were collected sufficiently intact for further analysis and were stored in 70% ethanol for subsequent dissection.

### Dissection

One antenna was removed from each male using forceps and scalpel. If the antenna had been damaged, the longest antenna was selected. The forewings, hindwings, and the head segment were also removed prior to measurement. The testes were collected by slicing open the abdomen with a scalpel and forceps, and removing the small dark-colored testis mass from the surrounding white tissue mass (the duplex) (Carpenter et al. 2009).

### Image capture and anatomical measurements

For each male specimen, high-resolution photographs of the antennae (coarse full structure scale and fine scale showing individual sensory hairs), testis, wings, and head were taken using a Leica MZ12 microscope and an auto-montaging camera. A scale bar was embedded in every image for subsequent digital image analysis and measurement using the freely available software ImageJ™ image analysis software package (Schneider et al. 2012).

**Figure 2:**
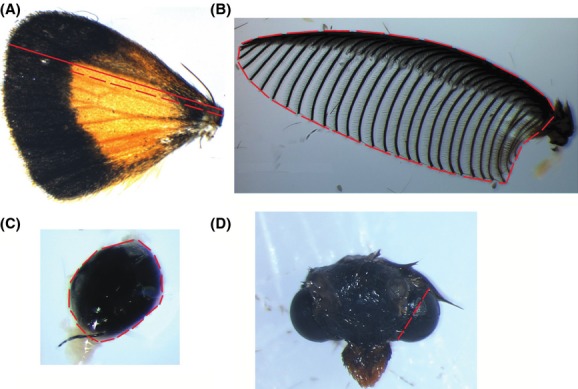
Measurements of morphological structures of *Teia anartoides*: (A) hindwing length and orange patch length, (B) antennal area, (C) testis area, (D) eye diameter.

Forewing size is commonly used as an indicator of overall body size in moths (Ingleby et al. 2010). However, the forewings of the majority of individuals obtained from the field traps were severely damaged, and so hindwing length (Fig.2A) was used to estimate body size. Analysis of laboratory-reared males revealed a strong correlation (*r* = 0.799, *n* = 31, *P* < 0.001) between forewing and hindwing length (B. Shiel, unpubl. results).

Antennal images were taken with the longer, flatter side of antennal branches resting against the supporting surface. Antennal area (mm^2^) was determined by measuring the polygonal area around the total span of one side of the antennal comb (Fig.2B). The head of each moth was photographed so that the position of the head and eyes were aligned and symmetrical. Eye diameter of each moth was determined at the widest span of the eye (Fig.2D).

Following Rogers et al. (2005), testis area was used as an estimate of testis size. The largest surface of the testis was exposed to the camera and we measured its polygonal area (Fig.2C). Due to damage incurred during dissection, we were unable to accurately assess testis area for two males, reducing our sample size for analyses using this trait to 49.

The exact size of the orange patch of each wing could not be calculated because the wing was often damaged during dissection (the lower portion of the hindwing is directly connected to the carapace of the abdomen and was often torn during wing extraction). Thus, we estimate patch size as the length of the orange color section along the same measurement line used for hindwing length (Fig.2A).

### Statistical analysis

Linear regressions were used to assess the relationships of body components (antenna area, eye diameter, and length of orange patch on hindwing) to body size (hindwing length). For estimation of allometric scaling exponents between morphological traits and body size, we log-transformed all measurements prior to this analysis. We used ordinary least-squares (OLS) regression, although we recognize the arguments surrounding whether type II (e.g., standardized major-axis - SMA) regressions, which do not assume absence of error in the *x* variable, are more appropriate (Warton et al. 2006). However, for simple allometric estimates, SMA regression is problematic and may give wrong estimates (for recent discussion see Voje et al. 2014), hence our choice of OLS regression.

Associations between antennal size, eye size, and orange patch size were analyzed using partial correlation, controlling for body size (hindwing length). We also analyzed a combined measure of “total sensory structures.” This value was the product of the standardized (raw value divided by the mean) values for antennal size and eye size. As testis size was not related to body size (see results), we used (standard) bivariate Pearson correlation tests for associations of testes size with the other variables (in this case, these yielded the same qualitative outcomes as partial correlation tests anyway). In all correlation tests, data were not transformed as they met the assumptions of normality, homogeneity, and linearity without such transformation. Statistical analyses were conducted using IBM SPSS™ statistics 20 (IBM SPSS Inc 2011).

## Results

**Figure 3:**
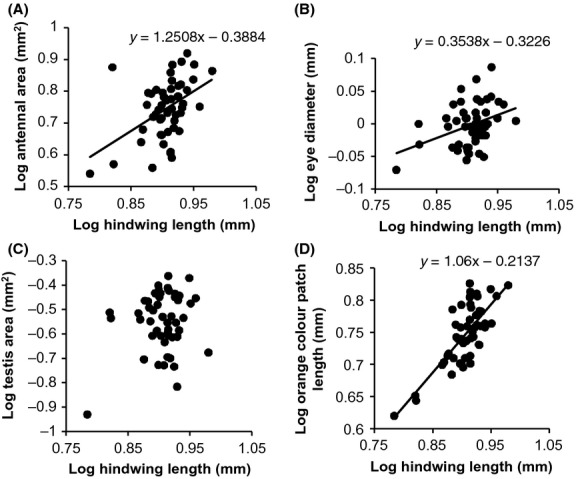
Allometric relationships between aspects of body morphology and body size (hindwing length) for *Teia anartoides*. Relationship shown for (A) antennal size, (B) eye size, (C) testis size, (D) wing orange patch size.

With the exception of testis area, morphological traits show strongly significant associations with body size (hindwing length) (Table1, Fig.3). The estimates for antennal area and orange patch size suggest slight positive allometry, but the isometric relationship (*β* = 1) lies within the error band of the estimates. Eye diameter exhibits negative allometry indicating that larger individuals have relatively smaller eyes (Table1).

**Table 1:** Allometric relationships between body components and hindwing length in the painted apple moth *Teia anartoides* (estimated using log-transformed measurements). Statistically significant *P* values (<0.05) are highlighted in bold

Component	*N*	Estimate	SE	*R* ^2^	*t*	*P*
Antennal area	51	1.251	0.345	0.196	3.626	**0.001**
Testis area	49	0.715	0.504	0.021	1.417	0.163
Orange length on hindwing	51	1.060	0.128	0.575	8.288	**<0.001**
Eye Diameter	51	0.354	0.128	0.117	2.756	**0.008**

**Figure 4:**
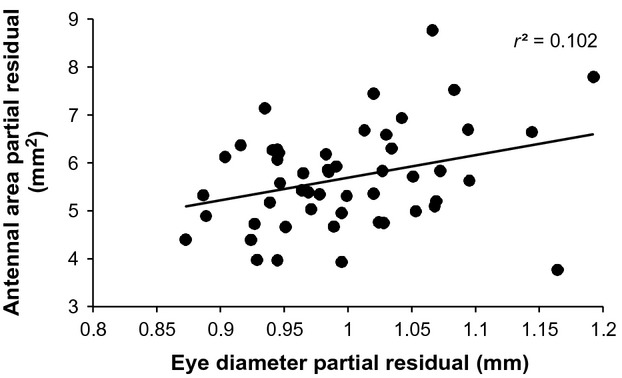
Relationship between relative eye size and relative antennal size in *Teia anartoides*. Partial residuals, controlling for body size (hindwing length) are plotted.

**Figure 5:**
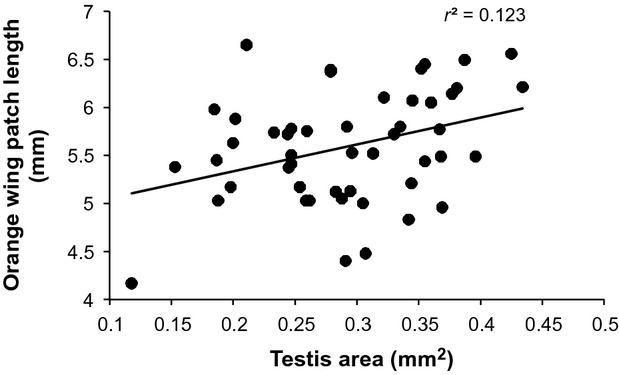
Relationship between testis size and size of orange patch on hindwings in *Teia anartoides*.

We found no significant evidence of trade-offs in the size of sensory structures (either individually or combined), testes, or orange color patches (Table2). By contrast, we found significant positive associations between two sets of traits: moths with larger eyes also had larger antennae, relative to body size (Fig.4); additionally, the size of the orange color patch on the hindwing was positively correlated with testis size (Fig.5). Although the latter result is not independent of hindwing size (i.e., is not a partial correlation), the lack of association between testis size and overall hindwing length suggests an association specifically with relative orange patch size, and not a surrogate estimate of body size.

**Table 2:** Correlation between morphological variables in the painted apple moth *Teia anartoides*. Values are partial correlation coefficients controlling for hindwing length, except for correlations with testis area. Statistically significant *P* values (<0.05) are highlighted in bold

	Antennal area	Eye diameter	Total sensory structures	Orange length on hindwing
Testis area				
*r*	0.169	0.261	0.214	0.351
df	48	48	48	47
*P*	0.240	0.068	0.135	**0.014**
Antennal area				
*r*		0.319	0.949	−0.178
df		48	48	48
*P*		**0.024**	**<0.001**	0.216
Eye diameter				
*r*			0.591	−0.013
df			48	48
*P*			**<0.001**	0.927
Total sensory structures				
*r*				−0.160
df				48
*P*				0.266

## Discussion

Reproductive strategies of males may require investment in either strategies for mate searching or securing paternity, or both. Larger males of *T. anartoides* have larger visual and olfactory sensory organs, but not larger testes, suggesting a selective advantage of large size in mate location but not paternity protection. As a monandrous species, this finding makes sense. In such species, testes size may not be under strong selection compared to species that exhibit higher levels of polyandry (Parker et al. 1997). Unsurprisingly, the size of orange patches on the hindwings in males is directly related to hindwing length, and scales isometrically, suggesting they are not a sexually selected signal (Bonduriansky 2007b). Intriguingly, we found evidence for negative allometry in eye size (smaller males have relatively larger eyes). Butterflies too, show slight negative allometry in eye size (Rutowksi 2000), which may suggest that certain minimal levels of optical acuity cannot be compromised in diurnal Lepidoptera.

In polyandrous species, if resources are limited, theory predicts a trade-off between traits associated with postcopulatory sexual selection (such as testis size) and traits that provide precopulatory advantages (such as mate location) (Simmons et al. 1999; Simmons and Emlen 2006). As predicted for a monandrous species, we found no evidence of such trade-offs in *T. anartoides*, as there was no significant negative correlations with testis size of any of the traits we investigated.

Life-history theory predicts a potential trade-off in investment in different elaborate ornaments and sensory structures, for example between antennae and eyes, because of the costs assumed to be associated with the production of such structures (Reznick 1985; Emlen 2001). However, evidence for costs associated with such structures is sometimes surprisingly absent. For example, McCullough and Emlen (2013) found that investment in the gigantic horns of the rhinoceros beetle, *Trypoxylus dichotomus*, appeared to have no detectable fitness cost associated with them, with no evidence for trade-offs with other structures. Furthermore, costs and associated trade-offs can be context-dependent and vary according to the specific environmental conditions (e.g., food availability, population density, ambient temperature) in which the animals are living (Sgro and Hoffmann 2004). Such differences may even lead changes in the direction of predicted relationships. Indeed, we found that, contrary to predictions, antennal size and eye size were significantly *positively* correlated in *T. anartoides*, even after controlling for body size. There are two other possible explanations for this surprising result. First, male reproductive success in this diurnally active, largely monogamous moth may depend critically on mate location, requiring investment in receptors that can detect both olfactory and visual cues. Individuals with greater eye size and antennal size may simply have greater capacity to extract resources from the environment (Van Noordwijk and de Jong 1986). The positive correlation may also reflect a mutual developmental history from the same imaginal structures (see Emlen and Allen 2003).

Wing color patterns in moths are typically viewed in the context of predator avoidance through crypsis, aposematism and mimicry (Endler 1984; Lindström et al. 2001; Moss et al. 2006; Nokelainen et al. 2012). While cryptic at rest, diurnally active male *T. anartoides* are highly vulnerable to predation when mate searching and mating. Consequently, if orange hindwing patches serve as a warning signal to predators, they may be regarded as a costly predator-avoidance trait that improves the likelihood of successfully locating a female. We found no evidence of a trade-off between this trait and sensory structures. By contrast, there is a positive correlation between testis size and orange patch size in *T. anartoides,* suggesting that these traits are linked. Although the effect is small (*r* = 0.351, equivalent to explaining *c*. 12% of the variation in testis size), this is close to the typical range for such correlational studies where a focal trait is related to a single other trait (Møller and Jennions 2002). Indeed, a meta-analysis of sperm traits in relation to male secondary sexual characteristics (Mautz et al. 2013) uncovered a statistically significant positive association across 38 studies, but with less than 1% of variation explained. Such traits may be phenotypically linked if they mutually reflect the stressors encountered at the larval stage (Kemp and Rutowsk 2007). Larval diet quality influences warning coloration in Lepidoptera (Lindstedt et al. 2010; Pegram et al. 2013), so the link between orange patch size and testis size may reflect general individual quality as a result of their larval rearing environment. The correlation may indicate shared genetic, developmental, or hormonal systems that determine investment in coloration and morphological characters (McKinnon and Pierotti 2010).

The lack of trade-offs between investment in secondary structures and between sperm production (testis size) and mate location traits cannot conclusively rule out differential investment. Ultimately, a limitation with correlational studies, such as this, is that it is impossible to determine whether genetic variability in the quality of individuals may be obscuring such trade-offs in investment (Van Noordwijk and de Jong 1986; Painting and Holwell 2013). However, we believe there is value in providing such data, particularly for monandrous species, where the predictions and patterns may differ from general expectations.
